# Assessment of the Impacts of Anthropogenic Activities on Woody Plant Diversity in the Woodlands of the Pette Subdivision (Far-North, Cameroon)

**DOI:** 10.1155/tswj/9974039

**Published:** 2024-12-24

**Authors:** Alphonse Diguera, Moksia Froumsia, Taffo Junior Baudoin Wouokoue, Dieudonne Jackba Danra

**Affiliations:** Department of Biological Sciences, University of Maroua, Maroua, Cameroon

**Keywords:** anthropogenic activities, floristic diversity, land cover, Landsat images, Pette

## Abstract

The land use/land cover in the Sudano-Sahelian area of Cameroon has been disturbed since these 3 decades resulting from the influence of anthropogenic factors. This study aimed to assess floristic diversity and the impacts of anthropogenic activities on the Pette forest massifs in the Pette Subdivision. The transect method (1000 × 20 m) was used for plant inventory, and Landsat images 5 TM (1990), 7 ETM+ (2005) and 8 OLI_TIRS (2020) were analysed to determine land cover. In total, 44 woody species belonging to 30 genera and 20 families were identified. The most frequent species (10.17%) was *Grewia bicolor*, and the species with high density was *Acacia ataxacantha* (56.87 stems·ha^−1^). The forest massifs were dominated by *Anogeissus leiocarpus* (36.01%), and the basal area was 234.77 m^2^·ha^−1^. The Shannon diversity index varies from 3.53 to 3.99 bits in Fadare and Tchakamadje forest massifs, respectively. The diameter and height distribution show an “L” shape reflecting juvenile vegetation. The analysis of Landsat images from 1990, 2005 and 2020 indicated an extension of agricultural lands (6234.54 ha in 1990 to 10,018.33 ha in 2020), bare soils/dwelling lands (14,049.90 ha in 1990 to 15,374.12 ha in 2020) and the degradation of shrub/tree savannah lands (74,312 ha in 1990 to 59,312.95 ha in 2020). The different forest massifs were rich, and anthropogenic activities were one of the main factors responsible for their degradation.

## 1. Introduction

In tropical regions, humans depend on the natural environment, which provides important products extracted from forests and savannah formations [1, 2]. The savannahs of the Sahelian area present substantive benefits for humans. It provides essential food, drugs and wood energy contributing to the well-being of populations [3]. Over these last decades, population growth has led to a drastic increase in the need for natural resources [4–6]. The growing awareness of the need for sustainable management of natural resources in the 1980s with the formulation of the concept of biodiversity and safeguarding of biodiversity remains a perpetual quest and a major concern beyond the provisions of the Rio de Janeiro World Summit on sustainable development. Despite these attempts to preserve natural environments, it remains difficult to find a consensus between the needs of populations and the safeguarding of biodiversity [7, 8]. The need for natural resources is becoming ever greater as the population increases in the Sub-Saharan area [9, 10]. However, savannah ecosystems have low productivity rates for nontimber forest products, which are far the basis of the socio-economic resources of many families in developing countries [8, 11–13]. In Far-North Cameroon, the population faces environmental challenges such as low rainfall, degradation of forested areas and increased drought. In addition to natural factors, there are anthropogenic factors such as agricultural activities, livestock breeding and wood cutting, which degrade the plant cover [14–16]. In the Sudano-Sahelian zone of Cameroon, these anthropogenic activities lead to a decrease in vegetation cover, biodiversity and the reduction of wooded areas [9, 17, 18]. However, there are still some wooded areas such as the forest massifs of Adoumere, Alagarno, Fadare and Tchakamadje in the Pette Subdivision, which provide wood energy, wood services, food and medicinal plants. Previous studies in the Far-North Region of Cameroon have been conducted on floristic diversity [19] along an Altitudinal Gradient in the Mount Maroua and anthropogenic impacts on the forest reserves [20–23], national parks [9] and plant uses [12, 24, 25]. Few studies have been carried out on the phytodiversity and impact of land uses on vegetation cover in the Pette Subdivision. It is therefore imperative to have reliable data to manage these areas. This study aimed to assess the floristic diversity and the impact of anthropogenic activities on the evolution of land use/land cover from 1990 to 2020.

## 2. Materials and Methods

### 2.1. Study Area

**Figure 1:**
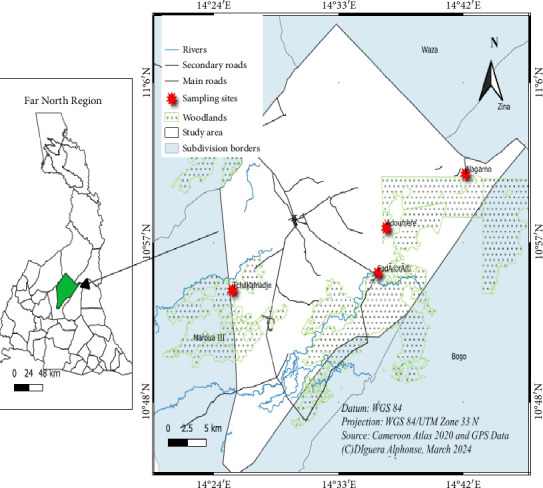
Map of the study area showing the location of different forest massifs.

The study has been conducted in four forest massifs (Alagarno, Adoumere, Fadare and Tchakamadje) located in the Pette Subdivision (10°47′–11°40′ north latitude and 14°25′–14°42′ east longitude), Diamare Division, Far-North Cameroon (Figure 1). This administrative unit covers an area of 700 km^2^. The climate is Sudano-Sahelian type with two seasons: the rainy season (from June to October) and the dry season (from November to May). The mean annual rainfall was less than 800 mm, and the average annual temperature was 30°C [9]. The soil was sandy on the dunes and at higher altitudes, clayey-sandy in the middle parts and essentially clayey in the lowlands. The dominant vegetation was tree savannahs, shrub savannahs and herbaceous savannahs.

### 2.2. Floristic Data Collection

Floristic inventories were carried out within four transects of 1000 × 20 m in each forest massif (16 transects in total). Five subplots of 20 × 20 m (80 subplots in total) were laid out within each transect to determine the dynamic of species renewal. The transects were laid out in different orientations from the centre of the forest massif, based on information gathered from field guides and preliminary observations. For this reason, orientations along the north-west, north-east, south-east and south-west axes were adopted for all collection units.

Diameter at breast height (DBH) was measured for tree individuals with a diameter ≥ 5 cm. The diameter classes with an amplitude of 5 cm have been established for the horizontal structure. The height was measured using a graduated pole. Height classes with an amplitude of 2 m have been established for the vertical structure. Cutting and dead individuals were also reported to calculate cutting and mortality rates, respectively.

### 2.3. Ethnobotanical Surveys

The ethnobotanical study was carried out near a population, involving 120 people aged between 35 and 75 years around forest massifs following surveys, using a pre-established questionnaire on local perceptions of degradation factors of forest massifs and the use of plants.

### 2.4. Estimation of Land Use and Land Cover Change

**Figure 2:**
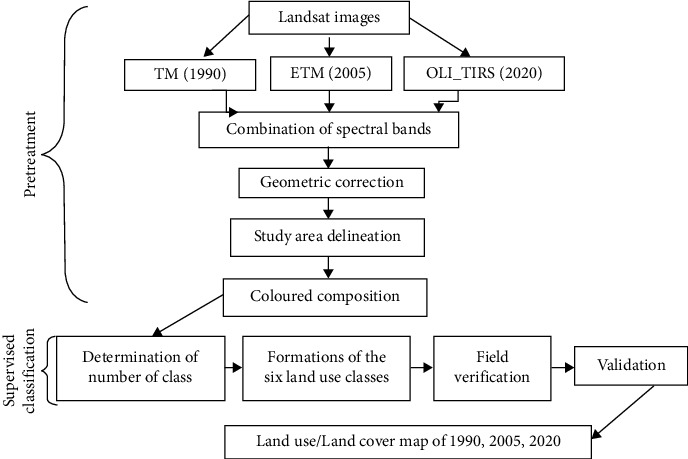
Images processing design.

The remote sensing consisted of analysing the Landsat 5 TM, Landsat 7 ETM+ and Landsat 8 OLI_TIRS satellite imagery of land use in 1990, 2005 and 2020, respectively with a spatial resolution of 30 m (Figure 2). These images contain location information provided by the National Geospatial Agency (NGA) and the USGS (US Geological Survey) and are freely downloadable. Geocover images have the advantage of being orthorectified Landsat images, so they can be easily integrated into a geographic information system (GIS). The digital image with a global resolution of 2.5 m was used to finalize the land use map. These images were acquired in the same period of the year (end of June) to ensure that the phenological stages of plant cover were not too different between dates. They are supplied in the standard Geotiff format with Universal Transverse Mercator (UTM) projection. The first phase of the treatment consisted of the combination of the different spectral bands to obtain the images of the three dates. The images used were already all georectified to UTM WGS84 with radiometric corrections. To avoid geographical deviation between images due to differences in the sensor when superimposing them for change detection analysis, the images of 1990 and 2005 were georectified to the image of 2020 already corrected using a ground survey. Using ERDAS Imagine, the enhanced false colour composite bands of the different years depicting the vegetation image pixels were trained and categorized into appropriate classes. After adjusting the image datasets, coloured compositions were created. Then, the study area was extracted from the scene to determine the land cover/land use types by supervised classification of images. We then carried out nondirected classifications that allowed us to segment the images into several subclasses of land use. These subclasses were grouped into six main classes: tree savannah, shrub savannah, bare soil, agricultural land, dwelling and water occupation, from comparisons based on the different spectral signatures of Landsat images [15]. The results from the classification were used to quantify the land cover and highlight the vegetation cover in 1990, 2005 and 2020. These treatments were carried out using ERDAS Imagine software. The images obtained were finally introduced into QGIS software for the realization of the map of land occupations of three dates.

### 2.5. Vegetation Composition and Structure Data Analysis

The following formulas were used to analyse the structural parameters:

(1) $$\begin{array}{c} \text{Basal area}\left(\text{Ba}\right):\text{Ba}=\pi \times \frac{{\text{Di}}^{2}}{4}\left(m^{2}{\text{ha}}^{-\left(1\right)}\right), \end{array}$$

where *π* = *C*/Di, where C is the circumference and Di is the diameter of each particular plant species measured at 1.30 m above the ground.

(2) $$\begin{array}{c} R\text{elative dominance}\left(\text{Rdo}\right):\text{Rdo}=\frac{\text{Ba}}{\Sigma \text{Ba}}\times 100\left(\%\right), \end{array}$$

(3) $$\begin{array}{c} \text{Relative density}\left(\text{Rd}\right):\text{Rd}=\frac{\text{Ni}}{N}\times 100\left(\%\right), \end{array}$$

where Ni is the number of individuals of a species and *N* is the number of individuals of all species.

(4) $$\begin{array}{c} \text{Relative frequency}\left(\text{Rf}\right):\text{Rf}=\frac{\text{fi}}{\Sigma \text{fi}}\times 100\left(\%\right), \end{array}$$

where (fi) is the frequency of a species *i*.

To describe the ecological importance of species and families in the sample area and of the woody flora of the massifs, the importance value index of families (family importance value, FIV) [26] and of species (importance value index, IVI) [27] were calculated as follows:

(5) $$\begin{array}{c} \text{Importance Value Index}\left(\text{IVI}\right):\text{IVI}=\text{Rdo}+\text{Rf}+\text{Rd}, \end{array}$$

where Rdo is the relative dominance, Rf is the relative frequency, Rd is the relative density;

(6) $$\begin{array}{c} \text{Family Importance value}\left(\text{FIV}\right):\text{FIV}=\text{Rdo}+\text{Rd}+\text{Rdi}, \end{array}$$

where Rdo is the relative dominance, Rd is the relative density, and Rdi is the relative diversity.

The theoretical value for which relative dominance, relative frequency, relative density and relative diversity belong is 0%–100% so that the IVI of a species and families can be between 0% and 300%.

(7) $$\begin{array}{c} \text{Renewal rate}\left(\text{Rr}\right):\text{Rr}=\frac{\text{Ns}}{N}\times 100, \end{array}$$

where Ns is the total number of seedlings and rejections and *N* is the total number count of individuals.

(8) $$\begin{array}{c} \text{Death rate}\left(\text{Dr}\right):\text{Dr}=\frac{\text{Nm}}{N}\times 100, \end{array}$$

where Nm is the number of death individuals in the population and *N* is the total number of individuals counted. It is the number of different species represented in an ecological community.

(9) $$\begin{array}{c} \text{Shannon}-\text{Weaver diversity index}\left(H^{\prime }\right):H^{\prime }=-\overset{s}{\underset{i=1}{\sum }}\text{pi}\times \log_{2}\left(\text{pi}\right), \end{array}$$

where Pi = (*ni*/*N*) × 100, ni is the number of individuals of species *i* and *N* is the total number of all individuals found.

(10) $$\begin{array}{c} \text{Pielou Evenness index}\left(\text{EQ}\right):\text{EQ}=\frac{H^{\prime }}{\log_{2}\left(S\right)}. \end{array}$$

EQ varies from 0 to 1.

(11) $$\begin{array}{c} \text{Simpson index}\left(D\right):D=1-\overset{s}{\underset{i=1}{\sum }}{\text{Pi}}^{2}. \end{array}$$

It measures the uncertainly that an individual taken randomly from the sample belongs to a given species.

P is the proportion (*n*/*N*) of individuals of one particular species found (*n*) divided by the total number of individuals found (*N*), Σ is the sum of the calculations, and *s* is the number of species. Diversity is high when its value is 0 and low for a value of 1.

(12) $$\begin{array}{c} \text{Sorensen index}\left(\text{Cs}\right):\text{Cs}=\frac{2c}{\left(a+b\right)}, \end{array}$$

With *a* being the number of species A, *b* being the number of species B and C being the number of common species for both zones.

### 2.6. Ethnobotanical Surveys

Ethnobotanical data processing was carried out using Excel 2019 software to calculate the sample size and relative frequency of citations,

(13) $$\begin{array}{c} \text{Sample size}\left(n\right):n=\frac{z^{2}\times p\left(1-p\right)}{m^{2}}, \end{array}$$

where *n* is the sample size, *z* is the confidence level (*z* = 1.96 for a confidence level of 95%), *p* is the estimated proportion of the population that has the characteristic and m is the margin of error tolerated (*m* = 5%).

(14) $$\begin{array}{c} \text{Relative frequency of citations}\left(\text{Rfc}\right):\text{Rfc}=\frac{S}{N\;}\times 100, \end{array}$$

where *S* is the number of people who responded to a given use and *N* is the total number of people interviewed.

### 2.7. Spatiotemporal Data

Spatiotemporal analysis was carried out by acquiring, classification and comparing Landsat images from the years 1990, 2005 and 2020,

(15) $$\begin{array}{c} \text{Annual expansion rate}\left(T\right):T=\frac{\left(\text{lnSr}-\text{lnSa}\right)}{\left(t*\text{lne}\right)}\times 100, \end{array}$$

with Sr being the surface of the recent year, *Sa* being the surface of the old year and *e* = 2.71828 [28].

## 3. Results and Discussion

### 3.1. Floristic Diversity

In total, 44 species belonging to 35 genera and 20 families were recorded in the Pette forest massifs, including 29 species in Fadare, 28 species in Adoumere, 23 species in Tchakamadje and 22 species in Alagarno (Table 1).

The most frequent species in different forest massifs were *Acacia ataxacantha* (6.15%) and *Balanites aegyptiaca* (6.15%) in Alagarno, *Acacia ataxacantha* (6.55%) and *Guiera senegalensis* (6.15%) in Adoumere, *Grewia bicolor* (6.15%) and *Sclerocarya birrea* (6.15%) in Fadare and *Grewia bicolor* (10.17%), *Acacia polyacantha* (6.90%) and *Anogeissus leiocarpus* (6.90%) in Tchakamadje (Table 2).

Species with high densities were *Acacia ataxacantha* (56.87 stems·ha^−1^) and *Balanites aegyptiaca* (20.62 stems·ha^−1^) in Fadare*, Ziziphus mauritiana* (32.25 stems·ha^−1^) and *Piliostigma reticulatum* (24.12 stems·ha^−1^) in Adoumere*, Acacia ataxacantha* (25.62 stems·ha^−1^) and *Balanites aegyptiaca* (21.87 stems·ha^−1^) in Alagarno and *Feretia apodanthera* (10.50 stems·ha^−1^) in Tchakamadje.

The highest total basal area was recorded in Alagarno (131.03 m^2^·ha^−1^) and the lowest in Tchakamadje forest massif (54.93 m^2^·ha^−1^). Species with high basal area were *Acacia sieberiana* (21.93 m^2^·ha^−1^) and *Sclerocarya birre*a (22.00 m^2^·ha^−1^) in Alagarno, *Balanites aegyptiaca* (17.21 m^2^·ha^−1^) and *Sclerocarya birrea* (13.61 m^2^·ha^−1^) in Fadare, *Balanites aegyptiaca* (15.00 m^2^·ha^−1^) and *Piliostigma reticulatum* (11.58 m^2^·ha^−1^) in Adoumere *Anogeissus leiocarpus* (19.78 m^2^·ha^−1^) and *Sclerocarya birrea* (11.92 m^2^·ha^−1^) in Tchakamadje.

The flora was dominated by *Sclerocarya birrea* (16.79%) in Fadare and Alagarno, *Piliostigma reticulatum* (10.84%) in Adoumere and *Anogeissus leiocarpus* (36.01%) in Tchakamadje. The highest importance value indices of species recorded were in the order *Balanites aegyptiaca* (86.42%), *Acacia ataxacantha* (67.16%), *Acacia sieberiana* (53.74%) and *Combretum aculeatum* (51.47%).

#### 3.1.1. Sorenson's Floristic Similarity Index

The floristic similarity index of Sorenson showed a value higher than 0.5 between the forest massifs. Adoumere and Alagarno were the most similar forest massifs with a Sorenson index of 0.76 (Table 3).

#### 3.1.2. Family Diversity

The densest families in different forest massifs were the Mimosaceae in Adoumere, Alagarno and Fadare with, respectively, 27.81%, 36.72% and 35.21% of relative density and Combretaceae (21.30%) in Tchakamadje (Table 4). The high relative diversity was recorded in Mimosaceae family with 17.86%, 37.82% and 17.24%, respectively, in Adoumere, Alagarno and Fadare, and Combretaceae (45.16%) in Tchakamadje. The families with high relative dominance were Mimosaceae with, respectively, 24.42% and 22.73% in Adoumere and Alagarno, Combretaceae with 5.13% in Fadare, and Mimosaceae and Combretaceae with 18.18% each in Tchakamadje. The Mimosaceae was the most important family with 70.09%, 97.27%, 57.00% and 42.01%, respectively, in Adoumere, Alagarno, Fadare and Tchakamadje forest massifs.

### 3.2. Horizontal Structure of the Vegetation

**Figure 3:**
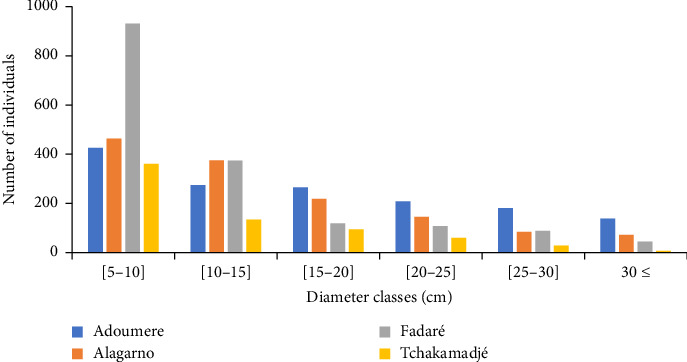
Horizontal structure of individuals.

The distribution of individuals in diameter classes showed an ‘L' shape structure. The great number of individuals was concentrated in the lower diameter classes ranging between 5–10 cm and 10–15 cm (Figure 3). Individuals with diameter class ≥ 30 cm were few in number in all forest massifs. Trees with large diameters were observed in species *Acacia albida*, *Celtis integrifolia*, *Sclerocarya birrea* and *Ficus ingens*.

### 3.3. Vertical Structure of Vegetation

**Figure 4:**
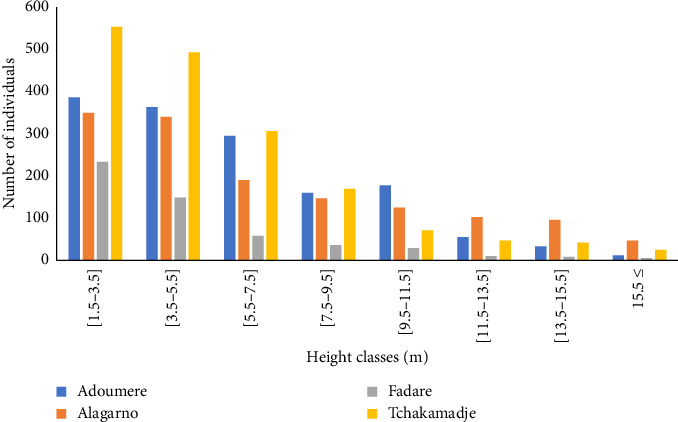
Vertical structure of individuals.

The height structure of the individual's analysis showed an “L” shape (Figure 4). The individuals were concentrated in lower classes 1.5–3.5 m and 3.5–5.5 m, and large trees were few in all forest massifs.

#### 3.3.1. Mortality rate

The global mortality rate was 11.16%; the highest rate was recorded in Tchakamadje (17.79%), and the lowest was 9.23% in Fadare (Table 5). Species with the high mortality rate were *Capparis sepiaria* (4.92%), *Acacia ataxacantha* (3.07%) and *Acacia gerardii* (3.03%).

#### 3.3.2. Renewal Rate

The mean regeneration rate was 23.64%. The best regeneration potential was recorded in Fadare forest massif (42.59%), followed by Alagarno, Tchakamadje and Adoumere with 26.08%, 21.46% and 4.44%, respectively (Table 6). Species with low regeneration rates were *Tamarindus indica* (0.14%), *Sclerocarya birrea* (0.70%), *Lannea fruticosa* (0.35%), *Grewia barteri* (0.42%), *Gardenia erubescens* (0.07%), *Diospyros mespiliformis* (0.28%) and *Dalbergia melanoxylon* (0.91%).

### 3.4. Perceptions of Vegetation Degradation of Land Cover by Local Community

**Figure 5:**
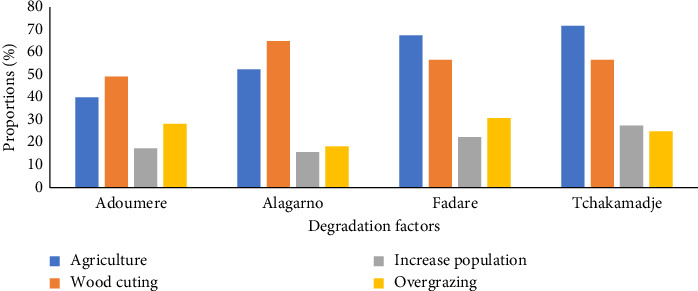
Proportions of main degradation factors.

Local people have knowledge of the land cover evolution, and more than 99% of respondents affirmed that the land has regressed during the period from 1990 to 2020 (Figure 5). This degradation is due to the agricultural activities by extension of cultural areas and wood cutting.

### 3.5. Evolution of the Land Cover and Land Use

**Figure 6:**
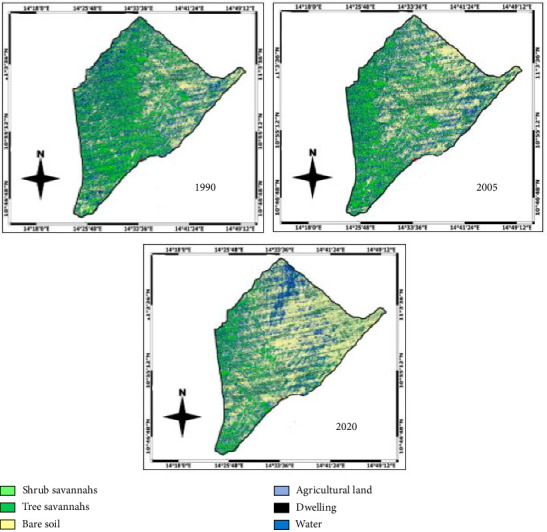
Maps of land occupation use of Pette Subdivision of 1990, 2005 and 2020.

The land use and land cover mapping identified six classes, namely, tree savannah, shrub savannah, agricultural area, water, bare soil and dwellings (Figure 6). Land cover statistics (Table 7) throughout the period of observation showed the dominance of tree savannah and shrub savannah.

### 3.6. Spatial Dynamics of Land Use Types From 1990 to 2020

**Figure 7:**
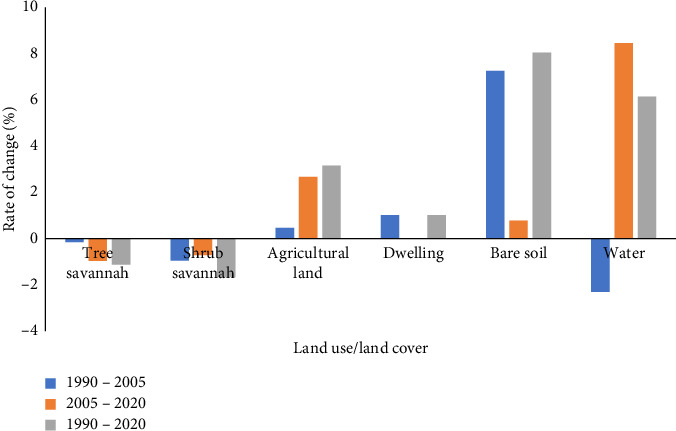
Annual rate of land change in Pette land use/land cover between 1990 and 2020.

From 1990 to 2020, the land use has undergone notable variations (Figure 7). There is a regressive dynamic noted in the areas of plant cover in favour of agricultural land, bare soil, water occupation and dwelling. The tree and shrub savannahs have lost 3350 and 10,649 ha, respectively. There has been an expansion of bare soil and agricultural areas as well as an increase in habitations during this period. The regeneration rate was 27.39%, the mortality rate was 11.16%, and the cutting rate was 23.43%.

## 4. Discussion

The recorded species richness was variable in all different forest massifs. These results were higher than 38 species obtained in the Sudano-Sahelian area by Jiagho, Banoho and Feumba [9]; Kaou et al. [29] and Todou et al. [24], respectively, in the Waza National Park, the dune zone in the south-east of Niger and the Moutourwa forest massif. However, the recorded number of species was less than 80 species obtained by Nangndi et al. [30] in the woody vegetation in the Sudano-Guinean zone of Larmanaye (Chad) and 86 species obtained by Froumsia [10] in the Kalfou Forest Reserve. According to Jhariya et al. [31], the differences observed could be due to the difference in anthropogenic pressures on studied vegetation such as wood cutting, agricultural activities, overgrazing and bushfires.

The high Shannon diversity indices could be explained by the high floristic diversity and the great number of plant species. The obtained values were higher than 1.56 bits obtained by Hamawa et al. [32] and lower than 4.92 and 4.82 bits, respectively, obtained by Junior Baudoin et al. [33] in Mount Mbapit savannah in the western highlands of Cameroon and Bakoulou, Souare and Ibrahim [34] in Kalfou Forest Reserve. This difference may be due to the intense anthropogenic activities in the studied area. The high evenness of Pielou and Simpson indices indicated a high equitability and high species diversity. These results were different to those found by Todou et al. [35] with a Simpson value index of 0.89. The high Sorenson floristic similarity index between the forest massifs of Adoumere and Alagarno could be explained by the low pressures of some anthropogenic activities such as wood cutting and overgrazing in the two forest massifs.

The dominance of *Anogeissus leiocarpus* (36.01%) was also observed by Hamawa et al. [32] who found that *Anogeissus leiocarpus* was the most dominant species in the Sahelian area of the Far-North Region, Cameroon. The greatest IVI (86.42%) recorded in this study was higher than those of *Guiera senegalensis* (66.76%) and *Sterculia setigera* (79.96%) obtained by Baïyabe II et al. [36] in the Sudano-Sahelian zone (Far-North, Cameroon). The richest families obtained in this study were similar to the results of Froumsia et al. [23] in the Sudano-Sahelian zones of North Cameroon. The most dominance of Mimosaceae could be due to their riches in species with individuals of high diameter sizes. Similar results were obtained by Froumsia [10] who also recorded the dominance of Mimosaceae in the reserve of Kalfou.

In all the sampled forest massifs, the diameters and heights of woody species showed a structure of an ‘L'shape curve. This structure could be due to the pressure anthropogenic activities such as wood collection (firewood, timber, etc.). The high proportion of smaller diameter and height stems in all forest massifs indicates a good regeneration. The high number of small trees within the shrub vegetation results in the great number of stems recorded in smaller diameter class 5–10 cm. This result was different from those of Froumsia [10] who found a high number of individuals in the highest diameter classes 20–30 cm. This difference could be due to the fact that their study was carried out in the protected zone. The high proportion of small-diameter individuals reassures that natural vegetation was ensured [37] and the low proportion of large-diameter individuals would be due to their overexploitation. According to Wouokoue et al. [38] and Kabre et al. [39], human activities through stumps shown from bushfires, wood cutting, overgrazing and other disturbance factors on vegetation disturbed biodiversity by affecting species diversity, their abundance and reducing the number of stems. The high proportion of individuals in lower height classes 1.5–3.5 m and 3.5–5.5 m observed in the height structure of the different forest massifs showed good natural regeneration and also indicates the rarity of large individuals due to the excessive cutting for wood energy. These results were similar to those recorded by Froumsia et al. [23], who found a high number of individuals in lower height class 3–6 m on the woodlands in the Sudano-Sahelian zones, North Cameroon, and 3–5 m recorded by Nangndi et al. [30] on the woody vegetation in the Sudano-Guinean zone of Larmanaye, Chad. The average regeneration rate obtained cannot be considered as a guarantee for renewal of the plant population of these forest massifs, given that young plants are frequently grazed by livestock and destroyed by bushfire. This rate was lower than that obtained by Diatta et al. [40] in the Ngazobil (Joal-Fadiouth) reserve in Senegal. According to Condit et al. [41], this low rate can be explained by the predation of seeds, viability, dormancy, and the dissemination mode that may be considered as factors influencing tree regeneration processes.

Landsat image analysis showed a regression of tree and shrub savannahs. The mortality rate combined with that of wood cutting was considerable, and added to agriculture, overgrazing and urbanization could be the anthropogenic factors at the origin of this change. The same observations were made by Tsewoue et al. [42] in Moungo, Littoral Region, Cameroon; Momo et al. [15] in Koup Matapit gallery forest, West-Cameroon; and Temgoua et al. [43] in Eastern Cameroon, indicating that anthropogenic activities such as logging and urbanization are the main factors of land use/land cover change. This change in land use/land cover leads to the disruption of ecosystems and the loss of biodiversity [44–47].

## 5. Conclusion

In Pette Subdivision, the forest massif cover is reducing despite its contribution to the daily life due to the increased needs of expanding population. This study showed that the different forest massifs were rich in species and diversified. The most abundant species was *Grewia bicolor*, the densest species was *Acacia ataxacantha*, and the high IVI was recorded in *Balanites aegyptiaca*. The most important families were Mimosaceae and Combretaceae. The distribution of individuals in diameter and height classes showed an ‘L' shape curve in all forest massifs. This structure may be due to the overexploitation of large-diameter individuals and the impact of agricultural activities. These factors were at the origin of changes in land use from 1990 to 2020 with a significant loss of wooded areas in favour of bare soil and dwellings. Given these results, it is important to involve the local population in the management of these forest massifs.

## Figures and Tables

**Table 1:** Floristic diversity of different forest massifs.

Forest massifs	Species	Genera	Families	Diversity indices		
				Shannon	Pielou	Simpson
Alagarno	22	18	12	3.96	0.86	0.08
Adoumere	28	24	16	3.67	0.74	0.10
Fadare	29	21	15	3.53	0.71	0.14
Tchakamadje	23	18	11	3.99	0.87	0.07

**Table 2:** Woody species, family, relative density (Rd), relative frequency (Rf%), relative dominance (Rdo%) and importance value index (IVI).

Species	Cronquist classification family	Forest massif															
		Adoumere				Alagarno				Fadare				Tchakamadje			
		Rd	Rf	Rdo	IVI	Rd	Rf	Rdo	IVI	Rd	Rf	Rdo	IVI	Rd	Rf	Rdo	IVI
	Mimosaceae	0.67	3.08	1.87	5.62	—	—	—	—	—	—	—	—	—	—	—	—
*Acacia ataxacantha*	Mimosaceae	5.93	6.55	4.12	16.20	15.92	6.15	4.50	26.57	26.44	5.56	2.56	34.56	—	—	—	—
*Acacia gerardii*	Mimosaceae	—	—	—	—	—	—	—	—	—	—	—	—	4.61	5.17	3.70	13.48
*Acacia hockii*	Mimosaceae	—	—	—	—	0.23	1.54	0.01	1.78	0.23	1.54	0.01	1.72	—	—	—	—
*Acacia nilotica*	Mimosaceae	1.08	3.08	0.15	4.30	—	—	—	—	1.86	2.78	0.36	5.00	—	—	—	—
*Acacia polyacantha*	Mimosaceae	—	—	—	—	4.35	4.62	2.03	10.99	2.09	4.17	2.83	9.09	3.66	6.90	2.93	13.48
*Acacia Senegal*	Mimosaceae	—	—	—	—	—	—	—	—	—	—	—	—	1.59	3.45	0.36	5.40
*Acacia seyal*	Mimosaceae	8.08	4.62	10.48	23.18	9.78	6.15	8.56	24.49	3.78	4.17	5.06	13.00	0.79	3.45	6.19	10.43
*Acacia sieberiana*	Mimosaceae	12.05	6.15	6.45	24.66	6.44	4.62	16.74	27.80	1.05	2.78	8.70	12.53	—	—	—	—
*Anogeissus leiocarpus*	Combretaceae	1.14	3.08	8.45	12.94	2.87	3.08	8.53	14.48	2.87	3.08	8.53	14.48	7.31	6.90	36.01	50.22
*Azadirachta indica*	Meliaceae	0.81	3.08	0.67	4.56	—	—	—	—	—	—	—	—	—	—	—	—
*Balanites aegyptiaca*	Balanitaceae	5.12	4.62	14.04	23.77	13.59	6.15	14.50	34.24	13.59	6.15	14.50	34.24	9.86	6.90	3.39	20.14
*Bauhinia rufescens*	Caesalpiniaceae	0.07	1.54	0.001	1.61	—	—	—	—	—	—	—	—	—	—	—	—
*Boscia senegalensis*	Capparaceae	0.94	3.08	0.39	4.41	4.43	4.62	0.89	9.93	4.43	4.62	0.89	9.93	10.65	6.90	3.66	21.21
*Calotropis procera*	Apocynaceae	0.54	1.54	0.01	2.08	—	—	—	—	—	—	—	—	—	—	—	—
*Capparis sepiaria*	Capparaceae	2.83	3.08	0.28	6.18	10.09	6.15	0.46	16.71	5.35	4.17	0.72	10.24	6.20	5.17	0.50	11.87
*Cassia sieberiana*	Fabaceae	—	—	—	—	—	—	—	—	—	—	—	—	2.70	3.45	0.03	6.18
*Celtis integrifolia*	Ulmaceae	0.13	1.54	0.13	1.80	1.16	3.08	0.08	4.32	1.16	3.08	0.08	4.32	—	—	—	—
*Cissus quadrangularis*	Vitaceaee	—	—	—	—	1.24	4.62	0.01	5.87	0.17	1.39	0.01	1.57	2.86	3.45	0.27	6.58
*Combretum aculeatum*	Combretaceae	2.22	4.62	8.64	15.48	3.11	4.62	6.87	14.59	2.96	5.56	11.60	20.12	7.47	5.17	7.66	20.31
*Combretum fragrans*	Combretaceae	—	—	—	—	—	—	—	—	0.46	2.78	0.63	3.87	—	—	—	—
*Combretum glutinosum*	Combretaceae	—	—	—	—	—	—	—	—	1.80	4.17	0.70	6.67	—	—	—	—
*Dalbergia melanoxylon*	Fabaceae	2.56	4.62	1.78	8.95	—	—	—	—	2.03	4.17	1.97	8.17	3.18	5.17	1.82	10.17
*Diospyros mespiliformis*	Ebenaceae	0.07	1.54	0.01	1.62	1.01	3.08	0.07	4.15	—	—	—	—	—	—	—	—
*Feretia apodanthera*	Rubiaceae	3.57	4.62	0.99	9.18	2.87	4.62	0.37	7.85	0.76	2.78	0.29	3.83	13.35	6.90	3.06	23.31
*Ficus ingens*	Moraceae	—	—	—	—	—	—	—	—	0.17	1.39	1.42	2.98	—	—	—	—
*Flueggea virosa*	Phyllanthaceae	0.54	3.08	0.01	3.62	1.71	4.62	0.01	6.33	1.34	4.17	0.03	5.53	1.11	1.72	0.01	2.85
*Gardenia erubescens*	Rubiaceae	—	—	—	—	—	—	—	—	0.41	2.78	0.08	3.26	—	—	—	—
*Gardenia ternifolia*	Rubiaceae	0.07	1.54	0.001	1.61	—	—	—	—	—	—	—	—	—	—	—	—
*Grewia barteri*	Malvaceae	—	—	—	—	—	—	—	—	0.87	2.78	0.17	3.83	—	—	—	—
*Grewia bicolor*	Malvaceae	0.81	3.08	0.11	4.00	3.34	6.15	0.23	6.72	3.34	6.15	0.23	9.72	6.90	10.17	1.17	18.24
*Grewia virosa*	Malvaceae	—	—	—	—	—	—	—	—	1.45	2.78	0.06	4.29	—	—	—	—
*Guiera senegalensis*	Combretaceae	17.10	6.15	4.75	28.01	4.89	4.62	0.41	9.92	4.69	4.62	0.41	9.92	5.17	6.52	1.49	13.18
*Lannea fruticosa*	Anacardiaceae	0.27	1.54	0.41	2.22	1.86	3.08	1.60	6.54	1.86	3.08	1.60	6.54	—	—	—	—
*Lannea velutina*	Anacardiaceae	—	—	—	—	—	—	—	—	—	—	—	—	1.72	0.48	0.05	2.25
*Mitragyna inermis*	Rubiaceae	1.41	3.08	10.42	14.91	2.95	4.62	13.74	21.30	2.95	4.62	13.74	21.30	—	—	—	—
*Piliostigma reticulatum*	Caesalpiniaceae	13.00	6.15	10.84	29.99	3.42	3.08	1.33	7.82	3.42	3.08	1.33	7.82	1.72	1.75	1.20	4.67
*Sclerocarya birrea*	Anacardiaceae	0.27	3.08	5.58	8.92	3.26	6.15	16.79	26.21	3.26	6.15	16.79	26.21	3.45	1.27	21.70	26.42
*Senna siamea*	Caesalpiniaceae	—	—	—	—	—	—	—	—	—	—	—	—	3.45	1.27	0.03	4.75
*Senna singueana*	Caesalpiniaceae	—	—	—	—	—	—	—	—	—	—	—	—	3.45	1.43	0.16	5.04
*Sterculia setigera*	Sterculiaceae	—	—	—	—	—	—	—	—	0.10	4.17	8.76	14.03	—	—	—	—
*Stereospermum kunthianum*	Bignoniaceae	—	—	—	—	—	—	—	—	0.17	1.39	0.07	1.63	—	—	—	—
*Tamarindus indica*	Caesalpiniaceae	0.67	3.08	2.15	5.90	1.48	4.62	2.28	8.37	0.23	1.39	0.07	1.63	3.45	1.75	4.61	9.80
*Ximenia americana*	Olacaceae	0.40	3.08	0.03	3.51	—	—	—	—	—	—	—	—	—	—	—	—
*Ziziphus mauritiana*	Rhamnaceae	17.37	6.15	7.24	30.77	—	—	—	—	2.09	4.17	1.21	7.47	—	—	—	—

**Table 3:** Sorenson floristic similarity index.

Massifs	Fadare	Adoumere	Alagarno	Tchakamadje
Fadare	1	0.59	0.66	0.69
Adoumere	0.59	1	0.76	0.54
Alagarno	0.66	0.76	1	0.75
Tchakamadje	0.69	0.54	0.75	1

**Table 4:** Forest massifs family, relative density (Rd), relative diversity (Rdi%), relative dominance (Rdo%) and family index value (FIV).

Family	Forest massif															
	Adoumere				Alagarno				Fadare				Tchakamadje			
	Rd	Rdi	Rdo	FIV	Rd	Rdi	Rdo	FIV	Rd	Rdi	Rdo	FIV	Rd	Rdi	Rdo	FIV
Anacardiaceae	0.54	7.14	0.47	8.15	5.12	9.09	21.84	36.06	1.39	3.35	3.57	8.41	1.75	21.74	9.09	32.58
Apocynaceae	0.54	3.57	0.01	4.12	—	—	—	—	—	—	—	—	—	—	—	—
Balanitaceae	5.12	3.57	14.86	23.55	13.59	4.55	17.23	35.36	9.59	3.45	4.51	17.55	9.86	3.38	4.54	17.78
Bignoniaceae	—	—	—	—	—	—	—	—	0.17	3.45	0.02	3.64	—	—	—	—
Capparaceae	3.77	7.14	0.71	11.62	14.52	9.09	1.61	25.22	5.69	6.90	0.22	12.81	16.85	4.15	9.09	30.09
Caesalpiniaceae	13.74	10.71	13.75	38.20	4.89	9.09	4.29	18.27	5.87	6.90	1.77	14.53	6.20	6.00	18.18	30.38
Combretaceae	20.74	10.71	23.12	54.58	10.87	13.64	0.001	24.51	28.70	13.79	5.13	47.63	21.30	45.16	13.63	80.10
Ebenaceae	0.07	3.57	0.01	3.65	1.01	4.55	0.08	5.64	—	—	—	—	—	—	—	—
Fabaceae	2.56	3.57	1.88	8.01	—	—	—	—	2.03	6.90	0.52	9.45	5.88	1.85	9.09	16.82
Malvaceae	0.81	3.57	0.12	4.50	3.34	4.55	0.27	8.16	4.30	10.34	0.14	14.79	10.17	1.16	4.54	15.88
Meliaceae	0.81	3.57	0.71	5.09	—	—	—	—	0.52	3.45	0.14	4.11	—	—	—	—
Mimosaceae	27.81	17.86	24.42	70.09	36.72	37.82	22.73	97.27	35.21	17.24	4.55	57.00	10.65	13.18	18.18	42.01
Moraceae	—	—	—	—	—	—	—	—	0.17	3.45	0.33	3.95	—	—	—	—
Olacaceae	0.40	3.57	0.04	4.01	—	—	—	—	—	—	—	—	—	—	—	—
Phyllanthaceae	0.54	3.57	0.01	4.12	1.71	4.55	0.01	6.25	1.34	3.45	0.01	4.79	1.11	0.01	4.54	5.67
Rhamnaceae	17.37	3.57	7.67	28.61					2.09	3.45	0.28	5.82	—	—	—	—
Rubiaceae	5.05	10.71	12.08	27.84	5.82	9.09	16.76	31.67	1.16	6.90	0.09	8.15	13.35	3.05	4.54	20.95
Sterculiaceae	—	—	—	—					1.10	3.45	2.04	6.69	—	—	—	—
Ulmaceae	0.13	3.57	0.14	3.84	1.16	4.55	0.10	5.81	—	—	—	—	—	—	—	—
Vitaceae	—	—	—	—	1.24	4.55	0.01	5.80	0.38	3.45	0.0007	3.82	2.86	0.27	4.54	7.68

**Table 5:** Mortality proportions of the five first species of each forest massif.

Species	Forest massifs			
	Adoumere	Alagarno	Fadare	Tchakamadje
*Acacia ataxacantha*	—	2.09	3.07	1.90
*Acacia gerrardii*	3.03	1.24	0.58	—
*Acacia seyal*	0.67	2.17	—	—
*Anogeissus leiocarpus*	—	—	0.81	5.08
*Balanites aegyptiaca*	—	—	—	1.58
*Boscia senegalensis*	—	0.93	—	1.27
*Capparis sepiaria*	1.34	1.31	1.85	4.92
*Guiera senegalensis*	1.07	—	0.58	—
*Piliostigma reticulatum*	1.14	—	—	—
*Ziziphus mauritiana*	—	—	—	1.90

**Table 6:** Renewal proportions of the five first species of each forest massif.

Species	Forest massifs			
	Adoumere	Alagarno	Fadare	Tchakamadje
*Acacia ataxacantha*	1.21	4.65	13.24	—
*Acacia seyal*	—	2.17	—	—
*Acacia sieberiana*	1.27	1.55	—	—
*Balanites aegyptiaca*	—	4.27	1.62	2.06
*Boscia senegalensis*	—	—	—	5.72
*Feretia apodanthera*	—	—	—	4.61
*Grewia bicolor*	—	—	—	1.58
*Guiera senegalensis*	2.82	—	14.17	—
*Piliostigma reticulatum*	2.08	—	2.78	—
*Ziziphus mauritiana*	4.44	—	—	—

**Table 7:** Areas and proportions of land cover types of 1990, 2005 and 2020.

Land use/land cover types	1990		2005		2020		T
	Area (ha)	%	Area (ha)	%	Area (ha)	%	
Tree savannah	21,654.03	24.42	21,154.07	23.86	18,304.10	20.64	−1.12
Shrub savannah	52,658.12	59.38	45,658.25	51.49	41,008.86	46.24	−1.66
Agricultural land	6234.54	7.03	6693.64	7.54	10,018.33	11.30	3.16
Dwelling	3006.86	3.40	3506.86	3.95	3506.85	3.96	1.02
Bare soil	3542.96	4.00	10,543.97	11.90	11,867.28	13.39	8.05
Water	1574.001	1.77	1114.12	1.26	3964.73	4.47	6.15
Total	88,670.51	100	88,670.91	100	88,670.15	100	/

*Note:* T: annual expansion.

## Data Availability

The data that support the findings of this study are available from the corresponding author upon reasonable request.
